# New diagnostic strategy for sepsis-induced disseminated intravascular coagulation: a prospective single-center observational study

**DOI:** 10.1186/cc13700

**Published:** 2014-01-20

**Authors:** Hiroyasu Ishikura, Takeshi Nishida, Akira Murai, Yoshihiko Nakamura, Yuhei Irie, Junichi Tanaka, Takehiro Umemura

**Affiliations:** Department of Emergency and Critical Care Medicine, Faculty of Medicine, Fukuoka University, 7-45-1 Nanakuma, Jonan-ku, Fukuoka, 814-0180 Japan

## Abstract

**Introduction:**

Inflammation and coagulation are closely interrelated pathophysiologic processes in the pathogenesis of sepsis. However, the diagnostic criteria of sepsis and disseminated intravascular coagulation (DIC) are different. This study aimed to define a biomarker panel to predict sepsis-induced DIC in emergency department patients.

**Methods:**

Eighty-two patients who were admitted to the emergency department of a tertiary university hospital were included in this study. The inclusion criteria were as follows: (1) age >18 years; (2) ≥1 systemic inflammatory response syndrome (SIRS) criteria. Patients were excluded if they lacked biomarker data or apparent clinical manifestations. Eleven biomarkers were assayed from blood drawn on ED admission. Receiver operating curve (ROC) analysis including the area under the ROC and multivariable logistic regression were used to identify an optimal combination of biomarkers to create a diagnostic panel. The derived formula for weighting biomarker values was used to determine the severity of sepsis-induced DIC, which was divided into three categories: mild, moderate, and severe. We also investigated the ability of this classification to predict secondary outcome measures of rates of sepsis and DIC, DIC score, acute physiology and chronic health evaluation (APACHE) II score, sequential organ failure score (SOFA) score, and 28-day all-cause mortality.

**Results:**

Among the 11 biomarkers tested, the optimal 2-marker panel comprised presepsin and protein C. The area under the curve for the accuracies of predicting sepsis and DIC from these two biomarkers were 0.913 and 0.880, respectively. When patients were divided according to the severity of sepsis-induced DIC, all secondary outcomes except for mortality were significantly higher depending on the severity (*P* < .0001). The overall mortality rates of mild, moderate, and severe sepsis-induced DIC were 7.14%, 15.4%, and 28.6%, respectively (*P* = .0994).

**Conclusions:**

A biomarker panel of presepsin and protein C is predictive of the severity of sepsis-induced DIC in suspected ED patients. These criteria for sepsis-induced DIC are very simple, easy to implement, and can be used in intensive care units as a point-of-care test.

## Introduction

Sepsis is often a complication that occurs in the clinical course of medical and surgical patients treated for other diseases [1, 2], and remains one of the most significant causes of mortality in intensive care units. The most recent international sepsis guidelines entitled “Surviving Sepsis Campaign: International Guidelines for Management of Severe Sepsis and Septic Shock: 2012” recommend early diagnosis and treatment of sepsis to avoid multiple organ failure and other adverse outcomes [3]. Sepsis is diagnosed based on evidence of infection along with the presence of systemic inflammatory response syndrome (SIRS) defined by the American College of Chest Physicians/Society of Critical Care Medicine (ACCP/SCCM) guidelines [4]. And sepsis was diagnosed when patients met the criteria for SIRS and an infectious source was documented or strongly suspected based on clinical presentation.

The majority of ill patients with SIRS present with coagulation abnormalities. Inflammation and coagulation play pivotal roles in the pathogenesis of sepsis. Evidence of extensive cross-talk between these two systems has been increasing [5]; inflammation leads to the activation of coagulation, which in turn considerably affects inflammatory activity. Since infection-induced disseminated intravascular coagulation (DIC) is closely associated with SIRS in a considerable percentage of patients, patients who exhibit two or more SIRS criteria for more than three consecutive days are frequently associated with DIC [6]. Sepsis is the most common disease associated with DIC. Approximately 20 to 40% of all sepsis patients are complicated with DIC [7–10]. There are some standard care procedures for sepsis, including the use of antibiotics, oxygen, fluid resuscitation and corticosteroids [11]. However, the mortality rate is still within the range of 30 to 50% in patients with septic shock [12, 13]. Gando *et al*. [6] reported that DIC is frequently associated with SIRS (83%) and that such patients have a high mortality rate (63%). Thus, the mortality rate of sepsis patients complicated with DIC is clearly higher than that of patients without DIC. Therefore, the early diagnosis and treatment of sepsis-induced DIC are critical for improving the prognosis.

However, there are different diagnostic systems and criteria for SIRS/sepsis and DIC. Furthermore, there are still no diagnostic criteria for sepsis-induced DIC, which may delay diagnosis and treatment initiation, and consequently be detrimental for the patient. Therefore, this study aimed to establish the diagnostic criteria for sepsis-induced DIC.

## Materials and methods

This prospective single-center observational study was conducted at the Department of Emergency and Critical Care Medicine, Fukuoka University Hospital, Fukuoka, Japan – a 915-bed referral, tertiary hospital - from June 2010 to June 2011. This study was approved by the institutional ethics committee, and all participants provided informed consent prior to participation. Patients aged ≥18 years who met one or more SIRS criteria were enrolled in this study. The background of these patients included liver cirrhosis, warfarin treatment, continuing antibiotics and/or steroid use, traumatic injury and others. We excluded patients who lacked a concentration of biomarkers or apparent clinical manifestations. Patients were evaluated for the presence of SIRS and sepsis according to the ACCP/SCCM guidelines [4]. The scoring system of the Japanese Association for Acute Medicine (JAAM) for DIC was used for the diagnosis of DIC in this study. This DIC diagnostic algorithm for scoring DIC includes the following variables: platelet count, prothrombin time, fibrin/fibrinogen degradation product level and SIRS criteria. The details of the algorithm have been published elsewhere [14]. DIC was defined by a score of ≥4. Illness severity was evaluated according to the Acute Physiology and Chronic Health Evaluation (APACHE) II score [15]. The APACHE II score assesses the illness severity of critical patients admitted to intensive care units on the basis of routine physiologic measurements, age and previous health status. It is used to predict the outcome of critical illnesses. Organ failure was assessed according to the Sequential Organ Failure Assessment (SOFA) score [16]. The SOFA score estimates organ dysfunction related to various disease statuses, especially sepsis, and is calculated using readily available measurements to quantify the dysfunction of the six major organs. Furthermore, it is useful for evaluating the morbidity and mortality of critical illnesses. All patients were followed-up for 28 days after enrollment in the study, and 28-day all-cause mortality was assessed.

### Study procedures

Blood samples for measuring the markers were collected on admission. Presepsin, procalcitonin (PCT), interleukin-6 (IL-6), C-reactive protein (CRP) and white blood cell (WBC) count were measured as inflammatory molecular markers in plasma. Antithrombin (AT), protein C (PC) activities, platelet count, prothrombin time (PT), D-dimer and thrombomodulin (TM) levels were measured as coagulation and fibrinolysis molecular markers. Platelet and WBC counts were measured in whole blood using an XT-1800i (Sysmex Co., Kobe, Japan). PT, D-dimer level, and PC and AT activities were measured in plasma using a Coapresta 2000 (Sekisui Medical, Tokyo, Japan). TM was measured using a STACIA (Mitsubishi Chemical Medience Corp., Tokyo, Japan). International normalized ratio (INR) was calculated using the following formula: INR = (patient PT/normal PT) × ISI, where normal PT represents the average of mean normal PT range of the laboratory result and ISI is the International Sensitivity Index, which is the correction coefficient of thromboplastin in commercial kits calculated according to international reference samples.

### Presepsin assay

Presepsin concentrations were measured using a compact automated immunoanalyzer, PATHFAST, based on a chemiluminescent enzyme immunoassay (CLEIA) (Mitsubishi Chemical Medience Corp., Japan) [17, 18]. Whole blood was collected using a conventional blood collection tube (TERUMO, Tokyo, Japan) with EDTA-2 K as an anticoagulant and used as a sample within 4 h after collection.

### PCT assay

PCT concentrations were measured by the Elecsys BRAHMS PCT assay (Roche Diagnostics, Tokyo, Japan) using EDTA plasma as a sample.

### Interleukin-6 assay

IL-6 concentrations were measured using the Immulyze 2000 assay system (Siemens Healthcare Diagnostics, Tokyo, Japan) using EDTA plasma as a sample.

### CRP assay

CRP concentrations were measured by CRP-LATEX (II) X2 “SEIKEN” (Denka Seiken Co., Ltd., Tokyo, Japan) using EDTA plasma as a sample.

### Statistical analysis

Unless otherwise indicated, all data are expressed as mean ± standard deviation (SD). SPSS 15.0 J (SPSS Inc., Chicago, IL, USA) was used for all statistical analyses. Comparisons between the two groups were made using unpaired Students *t*-test and either the χ^2^ test or Fisher’s exact test if necessary. Multiple datasets were analyzed by one-way ANOVA. The relationships between the measured variables and prognosis were analyzed by stepwise multiple logistic regression analysis with sepsis and/or DIC as the dependent variable. The results are reported as odds ratios (ORs) and 95% confidence intervals (95% CIs). Receiver-operating curve (ROC) analysis including the area under the ROC (AUC) was used to compare prognostic methods as predictors of sepsis and DIC. The standard error of the ROC was calculated using the formula based on Hanley and McNeil [19]. The level of significance was set at *P* <.05.

## Results

### Population characteristics

Of the 84 patients initially enrolled, we excluded 2 according to the exclusion criteria. Thus, a total of 82 patients were included in the analysis (Table 1). The mean age of the patients (44 men and 38 women) was 67.2 ± 17.3 years (median: 72.5 years, range: 21 to 93 years). Of all patients, 39 and 43 were diagnosed with non-sepsis and sepsis, respectively. In addition, 46 and 36 patients were classified into the non-DIC and DIC groups according to the JAAM DIC criteria, respectively. Furthermore, 26 patients with sepsis who met the JAAM DIC criteria were classified as having sepsis-induced DIC. The clinical characteristics of the 26 sepsis-induced DIC patients who met the sepsis and the JAAM DIC criteria were shown in Table 2.

**Table 1:** **Patient backgrounds**

Diagnosis	JAAM DIC		Total
	Negative	Positive	
Non-infection	8	4	12
SIRS	15	4	19
Infection	6	2	8
Sepsis	5	3	8
Severe sepsis	4	10	14
Septic shock	8	13	21
Total	46	36	82

DIC, disseminated intravascular coagulation; JAAM, Japanese Association for Acute Medicine; SIRS, systemic inflammatory response syndrome.

**Table 2:** **Clinical characteristics of the 26 sepsis-induced DIC patients**

Characteristics	Number of patients
Respiratory	10
Intra- or retro-abdominal, or pelvic cavity	9
Soft tissue or bone	4
Urinary	1
Blood or catheter	1
Unknown	1
Total	26

DIC, disseminated intravascular coagulation.

### Biomarker distributions

The means and SDs of coagulation and fibrinolysis molecular markers (AT and PC activities, platelet count, prothrombin time-international normalized ratio (PT-INR), and TM and D-dimer levels) and inflammatory molecular markers (presepsin, PCT, IL-6, CRP and WBC count) in patients with and without sepsis and DIC are shown in Tables 3 and 4, respectively. In addition, the AUC of each biomarker was calculated to evaluate the usefulness of each biomarker for the diagnoses of sepsis and DIC (Tables 3 and 4).

**Table 3:** **Biomarkers in the whole population and stratified by the presence or absence of sepsis**

Biomarker	Normal range	Overall population		Sepsis	
			Yes (n = 43)	No (n = 39)	ROC analysis
		Mean (SD)	Mean (SD)	Mean (SD)	AUC
**Coagulation and fibrinolysis molecular markers**					
AT activity, %	80 to 130	71.9 (28.1)	57.0 (23.9)	88.2 (23.4)**	0.828
PC activity, %	70 to 150	55.2 (34.6)	36.4 (24.5)	75.9 (32.7)**	0.834
TM, U/mL	<22	6.02 (6.19)	8.37 (7.91)	3.67 (2.00)**	0.802
Platelet counts, ×10^4^/μL	13.0 to 36.9	17.5 (10.7)	14.3 (8.73)	21.1 (11.8)**	0.681
PT-INR	0.85 to 1.15	1.63 (1.04)	1.85 (1.14)	1.40 (0.87)**	0.795
D-dimer, ng/mL	≦1.0	16.6 (17.4)	20.4 (17.8)	12.3 (16.4)*	0.659
**Inflammatory molecular markers**					
Presepsin, ×10^2^ pg/mL	<3.14	19.9 (36.2)	32.9 (46.2)	5.03 (4.64)**	0.887
PCT, ng/mL	<0.046	28.3 (66.9)	51.6 (85.6)	2.53 (11.8)**	0.904
IL-6, × 10^3^ pg/mL	<0.0034	36.9 (134.2 )	70.3 (179.8)	0.10 (0.13)**	0.893
CRP, mg/dL	≦0.2	9.88 (10.4 )	14.9 (10.5)	3.93 (6.73)**	0.852
WBC count, ×10^3^/μL	3.5 to 9.1	12.3 (6.94 )	12.3 (8.57)	12.3 (4.64)	0.503

AT, antithrombin; AUC, area under the curve; CRP, C-reactive protein; IL, interleukin; PC, protein C; PT-INR, prothrombin time-international normalized ratio; PTC, procalcitonin; ROC, receiver-operating curve; SD, standard deviation; TM, thrombomodulin; WBC, white blood cell.

*P <.05; **P <.01 vs. Sepsis group.

**Table 4:** **Biomarkers of the whole population and stratified according to the presence or absence of DIC**

Biomarker	Normal range	Overall population		DIC	
			Yes (n = 43)	No (n = 39)	ROC analysis
		Mean (SD)	Mean (SD)	Mean (SD)	AUC
**Coagulation and fibrinolysis molecular markers**					
AT activity, %	80 to 130	71.9 (28.1)	55.2 (22.5)	84.9 (25.2)**	0.807
PC activity, %	70 to 150	55.2 (34.6)	31.1 (18.8)	73.8 (32.4)**	0.877
TM, U/mL	<22	6.02 (6.19)	8.69 (8.61)	4.11 (2.16)**	0.730
Platelet counts, ×10^4^/μL	13.0 to 36.9	17.5 (10.7)	10.6 (6.45)	22.9 (10.4)**	0.859
PT-INR	0.85 to 1.15	1.63 (1.04)	1.83 (0.92)	1.48 (1.10)**	0.795
D -dimer, ng/mL	≦1.0	16.6 (17.4)	27.7 (18.3)	7.98 (10.6)**	0.844
**Inflammatory molecular markers**					
Presepsin, ×10^2^ pg/mL	<3.14	19.9 (36.2)	32.5 (44.4)	10.0 (24.5)**	0.808
PCT, ng/mL	<0.046	28.3 (66.9)	56.0 (92.1)	6.55 (18.9)**	0.785
IL-6, ×10^3^ pg/mL	<0.0034	36.9 (134.2 )	77.6 (195.7)	5.13 (16.7)**	0.765
CRP, mg/dL	≦0.2	9.88 (10.4 )	12.4 (10.7)	7.92 (9.93)*	0.650
WBC count, ×10^3^/μL	3.5 to 9.1	12.3 (6.94 )	11.7 (6.83)	12.8 (7.10)	0.528

AUC, area under the curve; AT, antithrombin; CRP, C-reactive protein; IL, interleukin; PC, protein C; PTC, procalcitonin; PT-INR, prothrombin time-international normalized ratio; ROC, receiver-operating curve; SD, standard deviation; TM, thrombomodulin; WBC, White blood cell.

*P <.05; **P <.01 vs. Sepsis group.

### Selection of a multi-marker panel and development of the sepsis-induced DIC diagnostic algorithm

In this study, biomarkers with an AUC of >0.8 were selected as part of the optimal biomarker panel (Tables 3 and 4). Regarding the inflammatory biomarkers, only the AUC for presepsin exceeded 0.8. Meanwhile, among the coagulation and fibrinolysis molecular markers, the AUCs of AT and PC were >0.8. The coagulation and fibrinolysis molecular markers to identify DIC patients with a primary outcome of DIC on admission were selected by multivariate logistic regression analysis using AT and PC. As a result, PC was the only statistically significant prognostic factor of DIC (OR: 0.939, 95% CI: 0.911 to 0.962; *P* < .0001). The following biomarkers were consequently found to compose the optimal biomarker panel: (1) presepsin (an inflammatory biomarker) and (2) PC (a coagulation marker). The AUCs for the combination of presepsin and PC in the patients with and without sepsis and DIC were 0.913 and 0.880, respectively.

**Figure 1:**
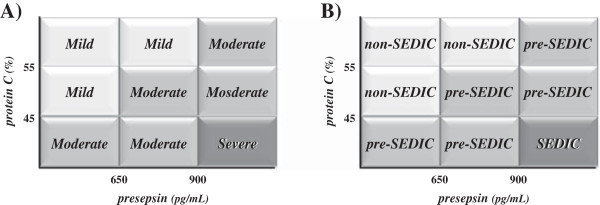
**Severity and classification of sepsis-induced DIC. A)** Severity of sepsis-induced DIC. Patients were classified into the following three groups according to presepsin and PC levels: (1) severe: presepsin >900 pg/mL and PC <45%; (2) mild: presepsin <650 pg/mL and PC >45%, or 650 < presepsin < 900 pg/mL and PC >55%; (3) moderate: parameters between the ranges of severe and mild. **B)** Classification of sepsis-induced DIC (SEDIC) criteria. The categories described in Figure 1A were classified as follows: (1) severe, SEDIC; (2) moderate, pre-SEDIC; (3) mild, non-SEDIC. DIC, disseminated intravascular coagulation; PC, protein C.

According to ROC analysis, the optimal cut-off values of presepsin and PC for the diagnosis of sepsis were 647 pg/mL and 47%, respectively; those for the diagnosis of DIC were 899 pg/mL and 55%, respectively (Table 5). The patients enrolled in this study were consequently divided into nine groups according to these cutoff values (Figure 1A).

**Table 5:** **Cut-off values of each biomarker stratified by the presence or absence of sepsis and DIC**

Biomarker		Sepsis			DIC	
	Cut-off value	Sensitivity	Specificity	Cut-off value	Sensitivity	Specificity
Presepsin, × 10^2^ pg/mL	647	93.0%	76.3%	899	83.3%	78.3%
PC activity, %	47	77.5%	81.1%	55	91.1%	72.7%

DIC, disseminated intravascular coagulation; PC, protein C.

Finally, we classified the patients into the following three groups according to presepsin concentration and PC activity: (1) severe (n = 28), presepsin >900 pg/mL and PC <45%; (2) mild (n = 28), presepsin <650 pg/mL and PC >45%, or 650 < presepsin <900 pg/mL and PC >55%; (3) moderate (n = 26), ranges between those of the severe and mild groups (Figure 1A).

The 28-day all-cause mortality of sepsis-induced DIC patients was significantly higher than that of patients who did not meet the criteria (Table 6). In this study, the sensitivity, specificity, positive predictive value and negative predictive value of the sepsis-induced DIC criteria were 80.7%, 87.5%, 90.7% and 75.0%, respectively (Table 7).

**Table 6:** **Comparison of 28-day all-cause mortality**

Diagnosis	28-day all-cause mortality		***P*** -value
	Negative	Positive	
JAAM DIC criteria	6.5% (3/46)	30.6% (11/36)	.0041
Sepsis criteria	7.7% (3/39)	25.6% (11/43)	.0316
JAAM DIC criteria + sepsis criteria	7.1% (4/56)	38.5% (10/26)	.0005

JAAM, Japanese Association for Acute Medicine; DIC, disseminated intravascular coagulation.

**Table 7:** **Sensitivity, specificity, positive predictive value and negative predictive value of sepsis-induced DIC criteria**

Diagnosis	Sensitivity	Specificity	Negative predicative value	Positive predicative value
Sepsis-induced DIC criteria	80.7% (21/26)	87.5% (49/56)	90.7% (21/28)	75.0% (49/54)

DIC, disseminated intravascular coagulation.

### Illness severity according to the classification of sepsis-induced DIC

We propose the sepsis-induced DIC diagnostic panel as a clinically useful numerical representation of the results from our multi-marker panel, which includes presepsin and PC panel testing. In addition, to evaluate the diagnostic accuracy of this panel, we investigated whether this panel was able to stratify patients according to the risks of sepsis and DIC as well as physiological severity, degree of organ failure and mortality (Table 8). When patients were classified according to sepsis-induced DIC severity, the severity increased significantly with increasing positive rates of sepsis and JAAM DIC. In addition, JAAM DIC, APACHE II and SOFA scores increased significantly with increasing severity (*P* <.0001) (Table 8). Significant differences in JAAM DIC, APACHE II and SOFA scores were observed between the mild and moderate groups (*P* <.0001) as well as between the mild and severe groups (*P* <.0001). Moreover, DIC and SOFA scores differed significantly between the moderate and severe groups (DIC *P* <.01; SOFA score *P* <.05).

**Table 8:** **Sepsis-induced DIC classification according to sepsis and DIC rates, diagnostic scores, and 28-day all-cause mortality**

Biomarker	Sepsis-induced DIC			***P*** -value
	Mild	Moderate	Severe	
	(n = 28)	(n = 26)	(n = 28)	
Sepsis (%)	10.7	61.5**	88.9**^#^	<.0001
JAAM DIC (%)	3.57	42.3**	85.7**^##^	<.0001
JAAM DIC score (SD)	1.61	3.46 (1.3)**	5.21 (1.7)**^##^	<.0001
APACHE II score (SD)	12.9	21.8 (7.8)**	23.5 (10.6)**	<.0001
SOFA score (SD)	3.64	8.00 (3.4)**	9.7 (3.5)**^#^	<.0001
28-day all-cause mortality (%)	7.14	15.4	28.6	0.0994

**P <.01 vs. Mild.

^#^ *P* <.05; ^##^ *P* <.01 vs. Moderate.

APACHE, Acute Physiology and Chronic Health Evaluation; DIC, disseminated intravascular coagulation; JAAM, Japanese Association for Acute Medicine; N.S., not significant; SD, standard deviation; SOFA, Sequential Organ Failure Assessment.

## Discussion

Sepsis is the most common cause of death in hospitalized patients and affects >18 million people worldwide; its incidence is expected to increase by 1% annually [20]. The mortality of patients who meet the severe sepsis criteria in the first 24 h after admission to the intensive care unit is 30 to 40% before intensive care unit discharge. Moreover, 45 to 50% of these patients die during their hospital stay [10, 12, 21]. In sepsis patients, bacterial products and cytokines also activate coagulation by increasing tissue factor (TF) synthesis and preventing fibrinolysis by increasing the level of Plasminogen Activator Inhibitor Type-1 (PAI-1). Sepsis is frequently complicated with DIC, which arises from fibrin accumulation in small vessels and occlusion of capillaries with microthrombi [22]. Activation of coagulation that arises from sepsis is accompanied by impaired function of major anticoagulant mechanisms including antithrombin, the PC tissue factor pathway inhibitor system and fibrinolysis [23, 24]. Pro-inflammatory cytokines and other mediators are capable of activating the coagulation system and down-regulating important physiological anticoagulant pathways [25]. These processes collectively result in increased levels of intravascular fibrin and the formation of microvascular thrombosis and, thus, ischemic multiple organ dysfunctions leading to necrosis [26].

DIC is associated with high mortality in patients with severe sepsis. The results of this study indicate the 28-day all-cause mortality of sepsis-induced DIC patients meeting the sepsis and JAAM DIC criteria was significantly higher than that of patients who did not meet these criteria (Table 6). Although the effectiveness of anticoagulant therapy in septic patients remains controversial, some studies suggest that rapid diagnosis and early treatment of DIC improve outcomes for these patients. In particular, therapeutic intervention directly against coagulation and inflammation in DIC associated with severe sepsis is effective [8, 27]. Furthermore, it is generally accepted that early aggressive treatment of the underlying disease is important.

In this study, we proposed new diagnostic criteria for sepsis-induced DIC. We assembled a cohort of patients with ≥1 SIRS criterion and investigated a series of biomarkers with the overall goal of creating a panel capable of diagnosing sepsis-induced DIC. Using an innovative approach to establish clinical utility, we created a panel of biomarkers that could provide clinicians with a tangible estimate of the increased risks of sepsis and DIC. The present results indicate that the optimal biomarkers for identifying sepsis-induced DIC are presepsin and PC, which represent inflammatory, and coagulation and fibrinolysis molecular markers, respectively. Presepsin and PC were obviously superior biomarkers for evaluating sepsis and DIC, respectively. This panel is biologically plausible as it incorporates biomarkers involved in key components of the pathophysiology of sepsis and DIC, including infection (presepsin) and activation of coagulation (PC). Moreover, we created new sepsis-induced DIC diagnostic criteria (Figure 1A), which included presepsin >900 pg/mL and PC <45%. This multi-marker approach offers a distinct mechanistic advantage over single-marker approaches.

Presepsin is a 13-kDa protein that is a truncated N-terminal fragment of CD14, the receptor for lipopolysaccharide (LPS)/LPS-binding protein (LBP) complexes [28, 29]. Its levels specifically increase in the blood of septic patients. The measurement of presepsin concentrations is reported to be useful for the diagnosis of sepsis, evaluating the severity of sepsis, and monitoring clinical responses to therapeutic interventions [30–34]. Most recently, multicenter clinical studies reported that presepsin is the most valuable predictive marker of sepsis between PCT and IL-6, and is superior to blood culture [35]. Our results corroborate the notion that presepsin is currently the most valuable predictive marker of sepsis. In addition, our results indicate that presepsin is the best predictive marker of DIC compared to other inflammatory molecular markers, including PCT, IL-6 and CRP.

Coagulation activation with subsequent diffuse intravascular fibrin deposition is implicated as an etiological factor in multiple organ dysfunction syndromes in patients with sepsis as well as in transplant and trauma patients [36]. Septic shock progression is associated with even greater mortality rates, ranging from 50% to 70% [37, 38]. The PC system plays a crucial role in the control of microvascular coagulation and inflammation; it is one of the basic regulatory systems of homeostasis, as it has potent anticoagulant, profibrinolytic and anti-inflammatory properties [39, 40]. PC is converted into activated PC (APC) under the formation of thrombin-thrombomodulin complexes with endothelial PC receptors (EPCRs) in the presence of protein S. Normal levels of circulating PC range from 2,800 to 5,600 ng/mL (80 to 140%), and the protein has a 10-h half-life. In contrast, APC has normal circulating levels from 1 to 3 ng/mL and a 20-minute half-life [39–42]. Activated PC inactivates coagulation factors Va and VIIIa and neutralizes the effects of PAI-l [43–45]. In addition, APC is capable of direct anti-inflammatory activity that reduces cytokine production (TNF, migration inhibitory factor (MIF)), thereby inhibiting the adhesion of leukocytes to the blood vessel endothelium [39, 40, 45]. As a result of all of the above mentioned mechanisms, APC significantly reduces the processes of microvascular thrombosis and endothelial dysfunction [46]. Numerous studies demonstrate depressed PC concentrations in both pediatric and adult septic patients are associated with increased morbidity and mortality [47–49]. The present study indicates that PC is more useful for evaluating DIC compared to other coagulation and fibrinolysis molecular markers.

These proposed criteria are useful for the diagnosis of sepsis-induced DIC with extremely high precision (AUC: approximately 0.9). Since the cutoff point for these criteria yielded the optimal sensitivity and specificity (80.7% and 87.5%, respectively), they were able to identify patients who likely developed sepsis-induced DIC. The severity of patients’ condition (DIC, APACHE II and SOFA scores) differed significantly not only between the mild and severe groups, but also between the mild and moderate groups. The severity of these criteria allows the differentiation of patients with respect to DIC severity, physiological illness (APACHE II), multiple organ failure (SOFA) and mortality. Besides the severe group, treatment should be ready to be initiated anytime in the moderate group.

In this study, we diagnosed DIC using the JAAM DIC diagnostic criteria. The JAAM DIC study group retrospectively analyzed patients with sepsis complicated with DIC (DIC diagnosed according to the JAAM DIC scoring system) using databases from two previous multicenter studies [14, 50]. They tested the hypothesis that the JAAM DIC scoring system constitutes a continuum dependent on the International Society on Thrombosis and Haemostasis (ISTH) overt DIC (DIC diagnosed according to the ISTH overt DIC scoring system) and that the JAAM DIC scoring system can predict full-blown DIC in a group of patients associated with systemic inflammation caused by infection. In other words, JAAM DIC with a stressed but compensated hemostatic system continuously progresses to ISTH overt DIC with a stressed but decompensated hemostatic system [51]. When JAAM DIC patients meet the ISTH overt DIC criteria, the risks of multiple organ dysfunction syndrome (MODS) and death are increased approximately 1.5 times. Therefore, the JAAM DIC scoring system is useful for determining patients with sepsis at a stage of stressed but compensated DIC [6].

This study has some limitations that should be noted. This study was a small, prospective, single-center, observational study. The present results suggest that there is no significant association between the severity of sepsis-induced DIC classification (severe, moderate and mild) and mortality. However, we have already initiated a validation study and planned a prospective multicenter study.

## Conclusion

The diagnostic criteria proposed herein are very simple, easy to employ and can be used in intensive care units as a point-of-care test. In addition, this scoring system is useful for the early treatment of sepsis-induced DIC in critical care settings. We also propose the new diagnostic criteria of sepsis-induced DIC termed “SEDIC” with the following categories: severe, SEDIC; moderate, pre-SEDIC; and mild, non-SEDIC (Figure 1B).

## Key messages

-  We proposed new diagnostic criteria for sepsis-induced DIC.
-  The optimal two-marker panel for sepsis-induced DIC comprises the following: (1) presepsin, an inflammatory biomarker and (2) protein C, a coagulation marker.
-  The new sepsis-induced DIC diagnostic criteria were defined as the following three groups: (1) severe, presepsin >900 pg/mL and PC <45%; (2) mild, presepsin <650 pg/mL and PC >45%, or 650 < presepsin <900 pg/mL and PC >55%; and (3) moderate, ranges between those of the severe and mild groups.
-  The severity of sepsis-induced DIC criteria proposed herein allows the discrimination of patients who have severe status according to DIC, physiological illness (APACHE II), multiple organ failure (SOFA) and mortality.

## Abbreviations

ACCP: American College of Chest Physicians
APACHE: Acute Physiology and Chronic Health Evaluation
APC: activated protein C
AT: antithrombin
AUC: area under the curve
CLEIA: chemiluminescent enzyme immunoassay
CRP: C-reactive protein
DIC: disseminated intravascular coagulation
ED: emergency department
IL-6: interleukin-6
INR: international normalized ratio
ISI: International Sensitivity Index
ISTH: International Society on Thrombosis and Haemostasis
JAAM: Japanese Association for Acute Medicine
PAI-1: Plasminogen Activator Inhibitor Type-1
PC: protein C
PCT: procalcitonin
PT: prothrombin time
ROC: receiver-operating curve
SCCM: Society of Critical Care Medicine
SD: standard deviation
SEDIC: sepsis-induced disseminated intravascular coagulation
SIRS: systemic inflammatory response syndrome
SOFA: sequential organ failure score
TM: thrombomodulin
WBC: White blood cell count.
